# The role of late gadolinium enhancement of the right ventricular insertion point predicts survival in patients with pulmonary hypertension

**DOI:** 10.1186/1532-429X-13-S1-O77

**Published:** 2011-02-02

**Authors:** Benjamin H Freed, Mardi Gomberg-Maitland, Sonal Chandra, Kim McCarty, Stuart Rich, Stephen Archer, Roberto MLang, Amit R Patel

**Affiliations:** 1 University of Chicago Medical Center, Chicago, IL, USA

## Objective

The aim of this study was to evaluate the relationship between late gadolinium enhancement (LGE) of the right ventricular (RV) insertion point (RVIP) and clinical outcomes in patients with pulmonary hypertension (PH).

## Background

Previous studies suggest a significant inverse correlation between extent of LGE and RV function. However, the potential role of LGE as a non-invasive predictor of survival remains unknown.

## Methods

We evaluated 83 consecutive patients with suspected PH referred for CMR between January 2009 and July 2010. Imaging was performed on a Philips 1.5T MRI scanner. Retrospectively gated cines of a short axis stack were obtained using SSFP (temporal resolution 25-40 ms). LGE images of the same views were obtained 10 minutes after infusion of Gd-DTPA (0.2 mmol/kg) using phase sensitive inversion recovery (TR 4.5 ms, TE 2.2 ms, TI 200-300 ms, flip angle 30°, PSIR flip angle 5°, voxel size 2x2x10mm, sense factor 2). The cines were used to determine RV volume, ejection fraction (RVEF) and mass. Two readers blinded to hemodynamic, functional, and laboratory data jointly determined the presence of RVIP-LGE. A subgroup of these patients underwent right heart catheterization (n=40) and exercise testing (n=54). Medical records and social security death index were reviewed on a monthly basis for occurrence of primary endpoint (hospitalization for PH exacerbation or all-cause mortality) in patients with cath-defined PH (mean pulmonary artery pressure [mPAP] > 25 mmHg).

## Results

**Figure 1:**
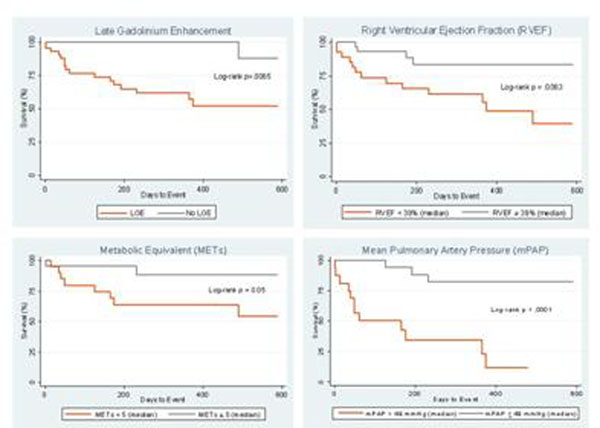


Overall, 45/78 (58%) of patients had RVIP-LGE. LGE was indeterminate in 5 patients. Patients with RVIP-LGE had a significantly larger RV volume and mass index, lower RVEF, higher PA pressure, lower cardiac index and lower metabolic equivalents (METs) achieved during treadmill test (Table 1. Patient Characteristics). Cath-defined PH was documented in 58/78 (74%) patients. Of these patients, 40/58 (69%) of patients had RVIP-LGE. During the mean follow-up period of 10.2 ± 6.3 months, 19 of these patients reached the primary endpoint. Using Cox proportional hazards modeling, LGE was a predictor for adverse outcomes (p = .026). In a multivariate analysis incorporating all significant univariate predictors, RVEF (p=.036), METs (p=.010), and mPAP (p=.001) remained independently associated with a poor prognosis (Figure 1. Kaplan-Meier curves).

**Table 1:** 

Cardiac Magnetic Resonance	All patients (n=78)	Patients with LGE (n=45)	Patients without LGE (n-33)	p value
RVEDVI (ml/m2)	118 ± 54	132 ± 56	94 ± 44	0.0001
RVESVI (ml/m2)	75 ± 52	89 ± 53	51 ± 42	0.0001
RVEF (%)	41 ± 14	36 ± 12	50 ± 12	<0.0001
RV Mass Index (g/m2)	24 ± 13	28 ± 14	17 ± 10	0.0002

**Naught on-Balke Exercise Treadmill Test**	All patients * (n=54)	**Patients with LGE (n=32)**	**Patients without LGE (n=22)**	**p value**

METs	6.2 ± 2.7	5.6 ± 2.5	7.3 ± 2.8	0.0245

**Right Heart Catheterization**	All patients* (n=40)	**Patients with LGE (n=28)**	**Patients without LGE (n=12)**	**p value**

Mean PA Pressure (mmHg)	41 ± 19	48 ± 18	29 ± 14	0.0002
Cardiac Indec (L/min/m2)	2.7 ± 0.95	2.4 ± 0.84	3.2 ± 1.1	0.0115

## Conclusions

In patients with PH, RVIP-LGE is associated with reduced RV function, poorer functional capacity, and worse hemodynamics. The presence of RVIP-LGE is a predictor of death and hospitalizations and may help identify patients who require earlier or more intensive medical management.

